# Latent class analysis of gambling subtypes and impulsive/compulsive associations: Time to rethink diagnostic boundaries for gambling disorder?

**DOI:** 10.1016/j.addbeh.2017.03.020

**Published:** 2017-09

**Authors:** Samuel R. Chamberlain, Jan Stochl, Sarah A. Redden, Brian L. Odlaug, Jon E. Grant

**Affiliations:** a Department of Psychiatry, University of Cambridge, UK; b Cambridge and Peterborough NHS Foundation Trust, UK; c Department of Psychiatry & Behavioral Neuroscience, University of Chicago, United States; d Department of Public Health, Faculty of Health and Medical Sciences, University of Copenhagen, Copenhagen, Denmark

**Keywords:** Addiction, Cognition, Gambling, Impulsivity, Latent, Subtypes

## Abstract

**Background:**

Gambling disorder has been associated with cognitive dysfunction and impaired quality of life. The current definition of non-pathological, problem, and pathological types of gambling is based on total symptom scores, which may overlook nuanced underlying presentations of gambling symptoms. The aims of the current study were (i) to identify subtypes of gambling in young adults, using latent class analysis, based on individual responses from the Structured Clinical Interview for Gambling Disorder (SCI-GD); and (ii) to explore relationships between these gambling subtypes, and clinical/cognitive measures.

**Methods:**

Total 582 non-treatment seeking young adults were recruited from two US cities, on the basis of gambling five or more times per year. Participants undertook clinical and neurocognitive assessment, including stop-signal, decision-making, and set-shifting tasks. Data from individual items of the Structured Clinical Interview for Gambling Disorder (SCI-GD) were entered into latent class analysis. Optimal number of classes representing gambling subtypes was identified using Bayesian Information Criterion and differences between them were explored using multivariate analysis of variance.

**Results:**

Three subtypes of gambling were identified, termed recreational gamblers (60.2% of the sample; reference group), problem gamblers (29.2%), and pathological gamblers (10.5%). Common quality of life impairment, elevated Barratt Impulsivity scores, occurrence of mainstream mental disorders, having a first degree relative with an addiction, and impaired decision-making were evident in both problem and pathological gambling groups. The diagnostic item ‘chasing losses’ most discriminated recreational from problem gamblers, while endorsement of ‘social, financial, or occupational losses due to gambling’ most discriminated pathological gambling from both other groups. Significantly higher rates of impulse control disorders occurred in the pathological group, versus the problem group, who in turn showed significantly higher rates than the reference group. The pathological group also had higher set-shifting errors and nicotine consumption.

**Conclusions:**

Even problem gamblers who had a relatively low total SCI-PG scores (mean endorsement of two items) exhibited impaired quality of life, objective cognitive impairment on decision-making, and occurrence of other mental disorders that did not differ significantly from those seen in the pathological gamblers. Furthermore, problem/pathological gambling was associated with other impulse control disorders, but not increased alcohol use. Groups differed on quality of life when classified using the data-driven approach, but not when classified using DSM cut-offs. Thus, the current DSM-5 approach will fail to discriminate a significant fraction of patients with biologically plausible, functionally impairing illness, and may not be ideal in terms of diagnostic classification. Cognitive distortions related to ‘chasing losses’ represent a particularly important candidate treatment target for early intervention.

## Highlights

• Current classification of gambling pathology relies on total number of criteria met for gambling disorder (DSM-5)
• This approach can be seen as somewhat arbitrary and may overlook meaningful gambling subtypes
• Latent class analysis (LCA) was used to identify candidate subtypes of gambling in 582 gamblers.
• Compared to the reference class, both problem gamblers and gambling disorder classes showed relatively impaired quality of life, and more psychiatric morbidity.
• Both problem gamblers and gambling disorder classes also showed higher impulsivity scores and impaired decision-making.
• LCA classification was sensitive to group quality of life differences, whereas DSM-5 classification was not.

## 1 Introduction

Gambling is a commonplace activity across cultures, and in extreme forms, can evolve into gambling disorder, a behavioral problem characterized by persistent, recurrent maladaptive patterns of gambling behavior and functional impairment. Lower levels of gambling pathology, however, have remained largely unexamined. For years, researchers and clinicians noted an intermediate level of gambling, termed “problem gambling”, which did not meet full diagnostic criteria but was associated with significant financial and personal difficulties (Currie et al., 2012). This lower level of gambling symptomatology, however, was never codified as a formal diagnosis.

Currently, the DSM-5 categorizes gambling disorder severity based on total symptom scores. For a diagnosis of gambling disorder, endorsement of four or more, out of nine criteria, is required (American Psychiatric Association, 2013). The DSM-5 definitions of disease severity are 4–5 criteria for mild, 6–7 for moderate, and 8–9 for severe gambling disorder. Problem gambling is not formally listed in DSM-5, but has been variably defined as endorsing two, or three, of the nine criteria in previous literature (Hodgins, Stea, & Grant, 2011). Existing definitions both for the disorder itself and for its severity levels are somewhat arbitrary: they are not necessarily based on meaningful subtypes, and may overlook underlying patterns in the distribution of diagnostic criteria endorsements. However, item-response analysis from a large dataset suggests that all the gambling disorder diagnostic criteria load onto one underlying dimension, and therefore that the sum can be used as a measure of severity (Strong & Kahler, 2007). Using classification and regression tree analysis, the diagnostic item related to preoccupation with gambling best distinguished social from problem gamblers in college athletes (Temcheff, Paskus, Potenza, & Derevensky, 2016).

Understanding of gambling ‘subtypes’ and how to classify people with gambling problems is highly relevant from neurobiological and clinical perspectives. Recent research suggests that selective cognitive dysfunction may already be present at lower levels of gambling pathology, even before individuals meet full criteria for gambling disorder. Specifically, Grant, Chamberlain, Schreiber, Odlaug, and Kim (2011) classified non pathological gamblers according to the total number of DSM criteria met, into two groups: social non-problem gamblers (zero diagnostic criteria met) and at-risk gamblers (1–2 diagnostic criteria met) (Grant et al., 2011). The at-risk gamblers, versus the control group, showed impaired performance on a computerized decision-making task – they gambled more points, made more irrational decisions, and were more likely to go bankrupt on the task. Decision-making deficits have commonly been reported in studies of patients with gambling disorder compared to healthy controls (Clark, 2010). Viewed collectively, these data suggest that some cognitive problems may exist not only in people with gambling disorder, but also in people with subthreshold symptoms.

It is important to consider whether quality of life might also be impaired in intermediate forms of gambling pathology. In a study using a large community-based sample, pathological gamblers had lower quality of life than problem gamblers, who in turn had lower quality of life than the non-problem gamblers (Scherrer et al., 2005). After adjustment for potential comorbidities, this group difference appeared to be specific for mental rather than physical health quality of life scores. In problem gamblers recruited from treatment programs, quality of life appeared to be worse in subtypes with psychological distress or multiple morbidities, as compared to subtypes with low comorbidities those with alcohol abuse (Suomi, Dowling, & Jackson, 2014). In a large study conducted in academic recruitment settings, quality of life was significantly different across controls, at-risk gamblers, and pathological gamblers (Loo, Shi, & Pu, 2016). The main effects of group on quality of life were significant and the at-risk gamblers were numerically intermediate between pathological gamblers and controls.

Understanding of underlying subtypes of gambling, with milder symptoms, may thereby allow for earlier interventions to thwart the development of gambling disorder with its potentially devastating consequences.

Latent class analysis represents a form of mixture modelling, whereby categorical responses on (for example) diagnostic questionnaires can be used to identify underlying latent subtypes in a data-driven fashion, such that individuals can be assigned to homogenous groups with similar symptom profiles (Goodman, 1974, Lazarsfeld and Henry, 1968). The technique has received only little application in gambling disorder research. In a study that drew data from two stratified surveys (n = 2417, and n = 530), at-risk gamblers and problem gamblers (defined using number of DSM-IV criteria) most commonly endorsed ‘chasing’ followed by ‘preoccupation and escape’ (Toce-Gerstein, Gerstein, & Volberg, 2003). ‘Withdrawal’ and ‘loss of control’ most distinguished pathological from problem gamblers. When the authors used regression modelling, they identified a latent dimension of gambling that was significantly linearly related to each individual gambling disorder criterion, excepting ‘chasing’ and ‘illegal acts’. A study of 3901 high school students, using latent class analysis with multiple health behaviors, classified adolescents into four classes: low-risk gambling (86.4%), at-risk chasing gambling (7.6%), at-risk negative consequences gambling (3.7%), and problem gambling (2.3%). At-risk and problem gambling groups were associated with greater negative functioning and more gambling behaviors (Kong et al., 2014).

In the case of adults, Carragher and McWilliams (2011) applied latent class analysis to the ten DSM-IV pathological gambling criteria using data from the National Epidemiologic Survey on Alcohol and Related Conditions (NESARC) (n = 11,104) (Carragher & McWilliams, 2011). They identified three latent classes based on gambling severity: no gambling problems (93.3%), moderate problems (6.1%) primarily endorsed the preoccupation, tolerance, and chasing criteria, and pervasive gambling problem (0.6%) endorsing the majority of the criteria. Similarly, McBride, Adamson, and Shevlin (2010) examined data from adults (n = 5644) who participated in the 2007 British Gambling Prevalence Survey and found three distinct classes of gamblers: non-problematic gamblers (88.9%); preoccupied chaser gamblers (9.7%); and antisocial impulsivist gamblers (1.4%) (McBride et al., 2010). Males, non-Whites and smokers were all more likely to be preoccupied chasers or antisocial impulsivist gamblers. Some of the work by McBride and colleagues has been further elaborated upon by James, O'Malley, and Tunney (2016). By examining multiple UK studies they argue that although there appear to be three classes based on gambling severity, there is evidence suggesting that intermediate and high severity disordered gamblers differed systematically in their responses to items related to loss of control, and not simply on likelihood of endorsing all diagnostic items equally (James et al., 2016).

These previous studies have been largely consistent showing that a small number of individuals qualify for severe gambling problems and that those who do usually endorse specific criteria (e.g., illegal behaviors) or often have related substance issues. Other studies examining personality traits and gambling symptomatology have found greater levels of sensation-seeking, high negative emotionality, and aggression in the more severe gambling (Savage et al., 2014, Studer et al., 2016). Elevated obsessive-compulsive traits have been reported in pathological gambling individuals compared to controls using a dimensional questionnaire (the Padua inventory) (Bottesi, Ghisi, Ouimet, Tira & Sanavio, 2015). In a latent class analysis study based on telephone interviews (participants from the Vietnam Era Twin Registry), participants were classified based on obsessive-compulsive (OC) symptoms (Scherrer, Xian, Slutske, Eisen, & Potenza, 2015). Four OC classes were identified: unaffected, rituals/symmetry, germs/fears, and severe. Compared to the unaffected class, the other classes had significantly higher endorsement rates of many individual pathological gambling criteria.

However, the available latent class modelling studies have not characterized whether different subtypes of gambling are associated with common or distinct neuropsychological profiles, which would be very informative from a neurobiological perspective. Furthermore, gambling disorder was regarded as an impulse control disorder in DSM-IV, yet the existing latent modelling studies largely did not screen for impulse control disorders, to evaluate comorbidity rates between subgroups. This issue is highly relevant since gambling disorder was moved from impulse control disorders to ‘Substance-Related and Addictive Disorders’ in DSM-5 (American Psychiatric Association, 2013).

The aims of the current study were (1) to identify subtypes of gambling in young adults, using latent class analysis, based on individual responses for the Structured Clinical Interview for Gambling Disorder (SCI-GD) for DSM-5; and (2) to explore how subtypes differed in terms of clinical presentation (including occurrence of impulse control disorders), and neuropsychological functioning. Based on existing literature, we hypothesized that three gambling types would emerge: recreational gambling, at risk or problem gambling, and gambling disorder. We further predicted that both the problem and pathological gambling groups would exhibit impaired decision-making, worse quality of life, and more obsessive-compulsive symptoms, than the reference group (Clark, 2010, Grant et al., 2011, Suomi et al., 2014, Scherrer et al., 2015, Loo et al., 2016).

## 2 Methods

### 2.1 Participants

Participants were recruited using media advertisements in two US cities, which asked “Do you gamble?” Inclusion criteria were age 18–29 years, being non-treatment seeking, and having gambled at least five times in the preceding year. Subjects were excluded if they were unable to give informed consent, or were unable understand/undertake the study procedures. This applied to no participants who attended for the research.

All study procedures were carried out in accordance with the Declaration of Helsinki. The Institutional Review Board of the University of Chicago approved the study and the consent statement. Participants were compensated with a $50 gift card for a local department store.

### 2.2 Clinical assessments

Gambling symptoms were evaluated using the Structured Clinical Interview for (SCI-GD) (Grant, Steinberg, Kim, Rounsaville, & Potenza, 2004). This instrument was originally designed to cover the DSM-IV diagnostic criteria. To make consistent with DSM-5 criteria, participant responses were recoded to remove the ‘illegal acts’ item.

The individual DSM-5 items covered by the SCI-GD are: gambling more over time, restlessness/irritability when cutting back, unsuccessful attempts to cut down on gambling, preoccupation with gambling, gambling during times of distress, chasing losses, hiding extent of gambling from others, risking job/education/career opportunities, and relying on others for money for gambling. By convention, full endorsement of four or more items on the SCI-GD would be consistent with gambling disorder, while fully endorsing 1–3 criteria would be considered problem gambling. Participants were also assessed for the frequency of gambling behavior as well as money lost gambling in the preceding year, using a timeline follow-back method for gambling (Weinstock, Whelan, & Meyers, 2004). The SCI-GD has excellent test-retest and inter-rater reliability (Grant et al., 2004).

Raters assessed each participant using the Mini-International Neuropsychiatric Interview (Sheehan et al., 1998), and the Minnesota Impulse Control Disorder (MIDI) screening tool (Grant, 2008); the former screens for mainstream mental disorders (e.g. depression, anxiety), while the latter screens for impulse control disorders (e.g. trichotillomania, kleptomania). Both instruments were designed as short, structured clinical interviews, for the assessment of mental disorders in research and clinical settings. Both have good to excellent test-retest and interrater reliability.

To assess personality-related impulsivity, participants also completed the Barratt Impulsivity Questionnaire (Patton et al., 1995, Stanford et al., 2016), which is a 30 item instrument that uses a 4-point scale for each response (responses are: rarely/never, occasionally, often, or almost always/always). The Barratt Questionnaire responses are used to generate three sub-scores on the basis of previous factor analysis: motor, non-planning, and attentional impulsivity. To assess obsessive-compulsive symptoms, we included the Padua inventory, a 60-item questionnaire originally developed to study obsessive-compulsive thoughts and behaviors in the general population (Sanavio, 1988). Each item is rated by the participant on a 5-point scale (not at all, a little, quite a lot, a lot, and very much). The Padua Inventory yields a total score but can also provide scores for specific OC symptom domains where further data exploration is indicated.

Quality of life was measured using the Quality of Life Inventory (QOLI) (Frisch et al., 1993), a self-complete questionnaire encompassing 16 domains that are important determinants of contentedness and life satisfaction. The QOLI contains 32 items, each with a 3-point rating for importance and 6-point scale for satisfaction. The QOLI yields a total score for overall quality of life, with lower scores equating to worse quality of life. It has good test-retest properties and has been validated against other measures including peer rating and clinical interview scores.

### 2.3 Cognitive assessments

Cognitive testing was undertaken using computerized paradigms from the Cambridge Neuropsychological Test Automated Battery (CANTAB). Participants completed cognitive testing in a quiet room, using a touch-screen computer. Based on the existing literature we focused on domains of decision-making, set-shifting, and response inhibition, which have been found to be impaired in people with gambling disorder versus controls (Clark, 2010, van Holst et al., 2010a, van Holst et al., 2010b, Grant et al., 2011). Decision-making was examined using the Cambridge Gamble Task (CGT) (Rogers et al., 1999). Participants were told that for each trial, the computer had hidden a ‘token’ inside one of ten boxes shown on the screen. These boxes were each either red or blue, and the participant indicated whether they felt the token would be hidden behind a red or a blue box. After making this judgment, participants gambled a proportion of their points on whether their color choice was correct. The key outcome measures were (i) mean proportion of points gambled; (ii) quality of decision-making (the proportion of trials where the volunteer chose red when red boxes were in the majority and vice versa – i.e. made the logical color choice); (iii) and risk adjustment (tendency to adjust how many points are gambled depending on the degree of risk).

We assessed response inhibition using the Stop-Signal Task (Aron, Robbins, & Poldrack, 2014), a paradigm in which the participant viewed a series of directional arrows appearing one per time on-screen, and made quick motor responses depending on the direction of each arrow (left button for a left-facing arrow, and vice versa). On a subset of trials, an auditory stop-signal occurred (a ‘beep’) to indicate that response suppression was needed for the given trial. The main outcome measure of the Stop-Signal Task is the stop-signal reaction time, which is an estimate of the time taken by the given volunteer's brain to suppress a response that would normally be undertaken.

Set-shifting was measured using the Intra-Dimensional/Extra-Dimensional Set-shift task (IED) (Pantelis et al., 1999). This task, derived from the Wisconsin Card Sorting Task, quantifies several aspects of rule learning and flexible behavior. Volunteers choose from two stimuli presented on the screen on each trial, and attempt to discover an underlying rule governing which stimulus is ‘correct’ (based on simple feedback provided by the computer). One the volunteer has learnt a given rule, the task then changes the rule. The main outcome measure on the task is the total number of errors made, adjusted for stages that were not attempted.

### 2.4 Data analysis

Data from all SCI-GD items were analyzed in poLCA (Linzer & Lewis, 2011) an R (R Development Core Team, 2011) package for polytomous latent class analysis. Fit of models with different number of classes was assessed using Bayesian Information Criterion (BIC) (Schwarz, 1978). The model with the lowest BIC was preferred.

Multivariate analysis of variance (MANOVA) was then used to explore clinical and cognitive differences between the identified gambling subtypes; because age differed between subtypes (see results), age was controlled for as a covariate in the models. Where the subtypes differed significantly on a given measure, least significant difference (LSD) tests were used to compare groups at the paired level. Statistical significance was defined as *p* < 0.05. MANOVA and LSD tests were undertaken using IBM SPSS Software, Version 22.

## 3 Results

The total sample comprised 581 individuals (mean age [SD] = 22.3 [3.6] years; 380 (65.4%) male). A three class latent model represented the optimal fit in terms of the Bayesian Information Criterion (BIC = 6840; versus 6926 for a two-class model, and 6904 for a four-class model). Entropy of the three class model was 0.82, indicating reasonable delineation of classes (Celeux & Soromenho, 1996).

**Fig. 1:**
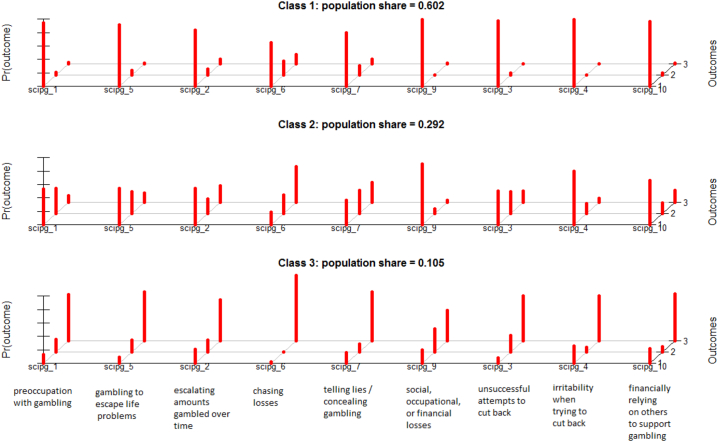
Distribution of individual SCI-GD responses in the three subgroups. Y-axis indicates the probability of a given individual in that subgroup endorsing each score on the item. 1 = criterion absent; 2 = criterion is partially met; 3 = criterion fully met. The item ‘chasing losses’ was particularly strongly endorsed in the problem gamblers group compared to the other items.

The profiles of SCI-GD endorsements associated with each class are shown in Fig. 1. The three classes were hereafter referred to as recreational gamblers (361 individuals, 62.1% of the sample), problem gamblers (161 individuals, 27.7% of the sample), and pathological gamblers (59 individuals, 10.2% of the sample) (note that percentages do not match population shares in Fig. 1, because Fig. 1 accounts for uncertainty of class allocation). It can be seen that ‘chasing losses’ was particularly strongly endorsed versus other diagnostic items in the problem gamblers group, whereas marked social, occupational, or financial losses item was rarely endorsed.

Demographic and clinical measures in the three classes are provided in Table 1. Groups differed overall on these parameters (MANOVA F = 15.042, *p* < 0.001). The recreational gamblers were younger than the problem gamblers, who in turn were younger than the pathological gamblers. The mean total number of diagnostic criteria endorsed for the three groups were 0.3, 1.9, and 6.4 respectively. Gender did not differ between subtypes, nor did rates of being in education or employment. Advancement of gambling symptoms was associated with incrementally lower proportion of White racial-ethnic status, higher occurrence of impulse control disorders, and more nicotine consumption. The problem and pathological groups showed significantly more occurrence of mainstream mental disorders and having a first-degree relative with an addiction, versus recreational gamblers, but did not differ significantly from each other on these measures. The pathological gamblers group lost more money to gambling than both other groups, who did not differ significantly from each other. Quality of life was impaired in problem and pathological gamblers versus recreational gamblers, and problem gamblers did not differ significantly from pathological gamblers on this measure.

**Table 1:** Demographic and clinical characteristics of gambling subtypes. Data are displayed as mean (standard deviation) or number of cases [% of group]. MANOVA refers to main effect of group. Post hoc tests were conducted when there was a main effect of group for a measure.

	Latent classification group			MANOVA, main effect of group		Post hoc tests		
	Mean (SD) or N [%]							
	Recreational gamblers (Rec) (*N* = 361)	Problem gamblers (Prob) (*N* = 161)	Pathological gamblers (Path)	F	p	RecvPath	RecvProb	ProbvPath
			(*N* = 59)					
Age, years	21.6 (3.4)	22.9 (3.6)	24.7 (3.2)	15.5	< 0.001	⁎⁎⁎	⁎⁎	⁎⁎
Sex male, n (%)	236 [65.4%]	103 [64.0%]	42 [71.2%]	0.07	0.937			
Ethnicity Caucasian, n [%]	301 [83.4%]	96 [59.6%]	31 [52.5%]	28.49	< 0.001	⁎⁎⁎	⁎⁎⁎	⁎⁎
Employed or enrolled in education, n [%]	326 [90.3%]	143 [88.9%]	43 [72.9%]	0.26	0.796			
Quality of life *t*-score	47.8 (10.3)	44.1 (12.0)	41.8 (16.7)	5.33	0.005	⁎⁎	⁎	ns
First-degree relative with an addiction, n [%]	81 [22.4%]	67 [41.6%]	34 [57.6%]	8.45	< 0.001	⁎⁎	⁎	ns
Presence of one or more mainstream mental disorders on MINI, n [%]	97 [26.9%]	77 [47.8%]	37 [62.7%]	12.09	< 0.001	⁎⁎	⁎⁎	ns
Presence of one or more impulse control disorders on MIDI, n [%] [besides gambling disorder]	9 [2.5%]	19 [11.8%]	10 [16.9%]	10.77	< 0.001	⁎⁎⁎	⁎	⁎⁎
SCI-GD total score (number of DSM-5 criteria met)	0.33 (0.56)	1.85 (1.24)	6.37 (1.53)	807.28	< 0.001	⁎⁎⁎	⁎⁎⁎	⁎⁎⁎
Amount lost to gambling past year, $	626.8 (3023.9)	1517.0 (3061.7)	5441.1 (7413.0)	21.34	< 0.001	⁎⁎⁎	ns	⁎⁎⁎
Number of times alcohol consumed per week	1.32 (1.40)	1.44 (1.44)	2.05 (1.80)	0.52	0.595			
Nicotine consumption, packs per day equivalent	0.08 (0.22)	0.17 (0.34)	0.30 (0.43)	8.94	< 0.001	⁎⁎⁎	⁎	⁎⁎

MINI = Mini International Neuropsychiatric Inventory; MIDI = Minnesota Impulse Disorder Inventory; SCI-GD = Structured Clinical Interview for Gambling Disorder. MANOVA: due to the significant group difference for age in the initial MANOVA, age was included as a covariate for the MANOVA data reported here for variables other than age. Post hoc tests are least significant difference (LSD) tests, ns non-significant.

For non-parametric variables or where normality was violated, the overall qualitative pattern of significant results was confirmed using equivalent non-parametric tests.

⁎ *p* < 0.05.

⁎⁎ *p* < 0.01.

⁎⁎⁎ *p* < 0.001.

Personality-related impulsivity scores, obsessive-compulsive symptom scores, and neurocognitive measures in the three classes are presented in Table 2. Groups differed overall on the personality-related impulsivity scores and neurocognitive parameters (MANOVA F = 5.082, *p* < 0.001). Both the pathological and problem gamblers groups showed elevated impulsivity on all three Barratt sub-scales compared to recreational gamblers, to a similar degree, while there was no main effect of group on Padua total scores. Compared to recreational gamblers, both problem and pathological gamblers bet more points, made worse decisions, and made less risk adjustment, on the Cambridge Gamble Task. Cognitive flexibility was significantly impaired only in the pathological gamblers versus recreational gamblers, whereas stop-signal inhibition was significantly impaired only in the problem gamblers. To check algorithm convergence on the Stop-Signal Task, we examined p(inhibit), which was close to 0.5 and did not differ significantly between the three groups (*p* > 0.10).

**Table 2:** Personality-related impulsivity, obsessive-compulsive symptoms, and cognitive characteristics of gambling subtypes. Data are displayed as mean (standard deviation) or number of cases [% of group]. MANOVA refers to main effect of group. Post hoc tests were conducted when there was a main effect of group for a measure.

	Latent classification group			MANOVA, main effect of group		Post hoc tests		
	Mean (SD) or N [%]							
	Recreational gamblers (Rec) (N = 361)	Problem gamblers (Prob) (N = 161)	Pathological gamblers (Path)	F	p	RecvPath	RecvProb	ProbvPath
			(*N* = 59)					
*Personality-related measures*								
Barratt attentional impulsivity	16.4 (3.9)	17.5 (4.2)	17.7 (4.2)	7.1	0.001	⁎	⁎⁎	ns
Barratt motor impulsivity	22.9 (4.4)	24.9 (4.6)	26.4 (5.3)	15.31	< 0.001	⁎⁎⁎	⁎⁎⁎	ns
Barratt non-planning impulsivity	23.3 (5.1)	25.4 (5.3)	26.5 (5.3)	12.61	< 0.001	⁎⁎⁎	⁎⁎⁎	ns
Padua compulsivity total score	17.1 (49.1)	20.6 (17.1)	34.5 (29.0)	2.56	0.078			

*Cognitive measures*								
CGT overall proportion of points bet	0.51 (0.14)	0.58 (0.13)	0.62 (0.12)	19.88	< 0.001	⁎⁎⁎	⁎⁎⁎	ns
CGT quality of decision-making	0.96 (0.08)	0.93 (0.10)	0.91 (0.09)	7.98	< 0.001	⁎⁎	⁎⁎	ns
CCT risk adjustment	1.84 (1.21)	1.156 (1.07)	0.79 (1.02)	22.77	< 0.001	⁎⁎⁎	⁎⁎⁎	ns
SST stop-signal reaction time, msec	176.0 (55.4)	192.8 (75.8)	188.0 (75.9)	2.57	0.077	ns	⁎	ns
IED total errors (adjusted)	9.4 (9.7)	10.7 (10.2)	15.0 (10.6)	5.86	0.003	⁎⁎	ns	⁎

Age was included as a covariate for the MANOVA data reported here. Post hoc tests are least significant difference (LSD) tests, ns non-significant.

For non-parametric variables or where normality was violated, the overall qualitative pattern of significant results was confirmed using equivalent non-parametric tests.

⁎ *p* < 0.05.

⁎⁎ *p* < 0.01.

⁎⁎⁎ *p* < 0.001.

Of the 360 participants classified as recreational gamblers by the latent class algorithm, 206 [57.2%] would have been defined as having no problem gambling by conventional DSM criteria, 131 [36.4%] as having problem gambling, and 23 [6.4%] as having gambling disorder. Of 159 participants classified by the algorithm as moderate risk gamblers, 66 [41.5%] would have been classified as having no problem gambling by conventional criteria, 82 [51.6%] as having problem gambling, and 11 [6.9%] as having gambling disorder. In the 59 participants classified as having severe gambling by the algorithm, 10 [16.9%] would have been classified as having no problem gambling by conventional criteria, 9 [15.3%] as having problem gambling, and 40 [67.8%] as having gambling disorder.

When study participants were grouped into no problem gambling, problem gambling, and gambling disorder categories based on conventional DSM criteria, there was no significant effect of group on quality of life (F = 2.018, *p* = 0.134). Post hoc paired tests confirmed that quality of life did not differ significantly between no problem gambling and gambling disorder cases, defined based on DSM criteria (*p* = 0.111).

## 4 Discussion

Using a large sample of non-treatment seeking young adults, enriched for gambling behavior, this study identified three distinct gambling classes based on Structured Clinical Interview for Gambling Disorder (SCI-GD) items: recreational gambling, problem gambling, and pathological gambling. We believe this to be the first latent class analysis that incorporated cognitive measures, to help address the biological validity or otherwise of the subgroups. Consistent with previous research in adult gamblers (McBride et al., 2010, Carragher and McWilliams, 2011), the high risk group was the least common, although our rates of 10.5% was notably higher than previous rates of 0.6% to 1.4%. We used the recreational gambling group as a reference point. The key findings were that (i) even problem gamblers who had a relatively low total SCI-PG score (mean endorsement of two items) had impaired quality of life to a similar extent as those with pathological gambling; (ii) problem and pathological gambling were associated with occurrence of mainstream mental disorders and impulse control disorders; (iii) the main diagnostic item serving to discriminate recreational from problem gamblers was endorsement of ‘chasing losses’; and (iv) decision-making deficits were common to problem and pathological gamblers. These findings have potentially important clinical, nosological, and neurobiological implications.

Because the problem gambling group showed impaired quality of life, but their symptoms had yet to progress to significant amounts of money being lost to gambling (Table 1), or much endorsement of social, occupational, or financial losses diagnostic item (Fig. 1), this group provides a valuable ‘window’ into factors that may be important in predisposing towards more severe gambling problems. Chasing losses refers to escalating one's gambling behavior (especially amounts gambled) after making a loss, which is one of the most commonly endorsed symptoms for gambling disorder (Toce-Gerstein et al., 2003). This phenomenon is related to the “gambler's fallacy”, in which gamblers perceive that a series of losses – or consistent outcomes (e.g. ‘red’ coming up in roulette) indicates that a reward or other outcome (e.g. ‘black’ coming up in roulette) is more likely (Studer et al., 2016). In our study, importantly, the diagnostic item most likely to discriminate recreational from problem gamblers was chasing losses (Fig. 1). Consistent with this finding, previous studies using latent class analysis of DSM criteria identified a ‘preoccupied chasing losses’ class of gamblers, which constituted an intermediate severity group (McBride et al., 2010, Kong et al., 2014). Similar results were reported using latent class analyses in conjunction with measures besides DSM criteria (Toce-Gerstein et al., 2003, Xian et al., 2008). Cognitive distortions related to chasing losses thus may constitute an important target for early intervention in people who are developing gambling problems. This issue could be via psychotherapy. Intriguingly, there is initial evidence that testosterone administration can reduce loss chasing in healthy women (Wu et al., 2016), raising the prospect of novel pharmacological strategies.

Problem and pathological gambling subtypes were both associated with higher occurrence of mainstream mental disorders and impulse control disorders, and higher personality-related measures of impulsiveness, but not with elevated obsessive-compulsive symptoms. It should be noted though that there was a trend towards an effect of group on Padua scores, due to increasingly high scores as a function of gambling severity group. Alcohol consumption did not differ significantly from recreational gamblers, whereas nicotine consumption was higher in the problem/pathological groups. These findings provide some support for gambling problems being conceptualized in terms of addiction, but also highlight that such symptoms might also be regarded as ‘impulsive’ in some ways. The mean number of DSM-5 items endorsed in the recreational, problem, and pathological gambling subtypes respectively were 0.3, 1.9, and 6.4. Individuals endorsing only 2 diagnostic criteria would fall well short of a diagnosis of gambling disorder and would not currently constitute a mental disorder by DSM-5 definitions, yet as a group they were impaired on quality of life to a similar degree as those with pathological gambling. At present, at least four DSM-5 criteria must be endorsed for a gambling disorder, and severity thresholds are 4–5 criteria for mild, 6–7 for moderate, and 8–9 for severe gambling disorder. We suggest that future nosological revisions should consider formally recognizing problem or ‘intermediate’ forms of maladaptive gambling symptoms.

From a biological perspective, problems with decision-making, set-shifting, and response inhibition have been commonly reported in people with gambling disorder (Clark, 2010, van Holst et al., 2010a, van Holst et al., 2010b, Grant et al., 2011). In previous work that classified participants based on the number of DSM items endorsed, using the same cognitive tests as used herein, problem gamblers showed decision-making deficits only, whereas other problems with response inhibition and set-shifting were found in pathological gamblers only (Grant et al., 2011, Odlaug et al., 2011). The current study is only partially consistent with this. Both problem and pathological gambler subtypes, compared to the reference group, had decision-making impairment. However, only the pathological gambler subgroup had set-shifting deficits, whereas only the problem gambler subgroup had response inhibition deficits. Viewed collectively, impaired decision-making appears to be a neurocognitive marker that is impaired even in milder manifestations of maladaptive gambling, suggesting that – as for ‘chasing losses’ – it could constitute a fruitful novel treatment target. Common deficits in decision-making in the problem and at risk latent class groups supports the biological plausibility of this conceptualization. This task is dependent on integrity of the orbitofrontal cortices and insula, regions implicated as functioning abnormally in gambling disorder (Clark, 2010, van Holst et al., 2010a, van Holst et al., 2010b).

There are several limitations to this study. The cross-sectional nature of the data prevents us from assessing – directly – the temporal element of gambling symptoms and clinical and cognitive findings. Future studies should examine gambling symptoms and various clinical and cognitive findings using longitudinal designs. Some of our participants were under the age of 21 years. As young adults reach the legal gambling age, their gambling behaviors may change because they have legal access to age-restricted venues and the patterns of associations with gambling-related and cognitive variables may also change over time.

The strength of this study is the large sample size of non-treatment seeking young adults using latent class analysis to identify subtypes of gambling based on the Structured Clinical Interview for Gambling Disorder (SCI-G). This method allows for a more detailed understanding of gambling symptomatology and allows for the development and understanding of less severe forms of illness. This in turn has the potential to drive better primary interventions. Future work should extend the current latent class analytic approach into clinical settings, as the current study focused on non-treatment seeking individuals. This may ultimately contribute to more meaningful or clinically helpful ways of subclassifying patients for the purposes of treatment selection or prioritization.

## Conflicts of interest

Dr. Chamberlain consults for Cambridge Cognition. Dr. Grant has received research grants from NIMH, National Center for Responsible Gaming, and Forest and Roche Pharmaceuticals. He receives yearly compensation from Springer Publishing for acting as Editor-in-Chief of the Journal of Gambling Studies and has received royalties from Oxford University Press, American Psychiatric Publishing, Inc., Norton Press, and McGraw Hill.

## Contributors

Samuel Chamberlain: Dr. Chamberlain undertook statistical analyses and drafted the manuscript.

Jan Stochl: Dr. Stochl undertook statistical analyses and assisted in drafting the manuscript.

Sarah Redden: Ms. Redden collected the data and assisted in drafting the manuscript.

Brian Odlaug: Dr. Odlaug collected the data and assisted in drafting the manuscript.

Jon Grant: Dr. Grant designed the study, collected the data and drafted the manuscript.
